# Genomic correlates of tailocin sensitivity in *Pseudomonas syringae*

**DOI:** 10.1093/g3journal/jkaf203

**Published:** 2025-08-29

**Authors:** David A Baltrus, Savannah Weaver, Laura Krings, Anh Evy Nguyen

**Affiliations:** School of Plant Sciences, The University of Arizona, Tucson, AZ 85721, United States; School of Animal and Comparative Biomedical Sciences, The University of Arizona, Tucson, AZ 85721, United States; Department of Molecular and Cellular Biology, The University of Arizona, Tucson, AZ 85721, United States; School of Plant Sciences, The University of Arizona, Tucson, AZ 85721, United States; School of Plant Sciences, The University of Arizona, Tucson, AZ 85721, United States

**Keywords:** phage-derived bacteriocin, *Pseudomonas syringae*, tailocin, lipopolysaccharide

## Abstract

Phage-derived bacteriocins, also referred to as tailocins, are structures encoded by bacterial genomes and deployed into the extracellular environment to kill sensitive cells. Tailocins display great potential as agricultural antimicrobials due to their durability, efficiency, and specificity of killing with prophylactic application demonstrated to prevent infection by multiple phytopathogens. Previous reports suggest that tailocins of *Pseudomonas syringae* interact with sugar moieties in the lipopolysaccharide (LPS) to target sensitive cells. However, it remains unclear how genetic and genomic variation at loci encoding LPS biosynthesis influences tailocin resistance and/or sensitivity across the species. We therefore carried out a genome-wide association study investigating tailocin sensitivity across a diverse set of *P. syringae* genomes. Our results demonstrate that genes strongly correlated with tailocin sensitivity are localized to one contiguous region on the chromosome encoding LPS structures similar to the common polysaccharide antigen of *P. aeruginosa*. We further find that enzymes involved in the biosynthesis and transport of D-rhamnose and L-rhamnose are associated with tailocin sensitivity classes A and B, respectively, with large-scale recombination of the *O*-antigen biosynthesis region likely underlying rapid and fundamental changes in LPS structure between strains. Building on these results, we identify *rfbD* as a genomic indicator for predicting tailocin sensitivity and use this information to test tailocin interactions with previously unscreened strains, including some in which LPS chains have been characterized. Overall, our results strongly support that tailocin sensitivity for *P. syringae* is broadly determined by recombination events across strains that leads to differential production of either d or L-rhamnose moieties in the main *O*-antigen chain.

## Teaser

Given worries about off-target effects of broad spectrum antibiotic use, there is great interest in developing new and strain-specific antimicrobial in agriculture. Although phage derived bacteriocins (tailocins) show exceptional potential for targeted killing of strains of interest and for prophylactic use to prevent infection by phytopathogens, we know relatively little about the genetic and genomic basis of resistance to tailocins across strains. Using genome wide association methods, Baltrus *et al*. identify a ~20Kb locus in the *Pseudomonas syringae* chromosome which is strongly correlated with tailocin sensitivity/resistance and demonstrate that the rfbD locus itself is a useful genomic marker of tailocin sensitivity.

## Introduction

Phage-derived bacteriocins, hereafter referred to as tailocins, are proteinaceous structures encoded by bacterial genomes thought to be used by strains in an extracellular manner to target and kill competing strains under natural conditions (Ghequire and De Mot 2015; Scholl 2017; Patz et al. 2019; Backman, Burbano, et al. 2024). The efficacy of killing, durability, and strain specificity of tailocins have made them exceptional candidates for the development and deployment as antimicrobials in clinical and agricultural environments (Príncipe et al. 2018; Baltrus et al. 2022). To best maximize the efficiency of use and develop methods to optimize application strategies, as well as to predict sensitivity of untested strains, we must better understand the genetic basis of resistance to tailocins across diverse suites of strains. Here, we present a genome-wide association analysis of loci that affect tailocin-based killing within *Pseudomonas syringae* strains and use this information to predict tailocin-based sensitivity for additional strains from their genome sequences.

Tailocins are part of a broad class of structures, also including type VI secretion systems and extracellular contractile injection systems (eCIS), co-opted by bacterial genomes from phages and structurally resembling phage tails (Scholl 2017; Patz et al. 2019). They are produced by bacterial cells and released through cell lysis, but unlike either the type VI systems or eCIS, they are not currently known to deliver effector proteins to target cells. Tailocins have independently and repeatedly evolved across taxa, with multiple types of rigid (R-type) tailocins evolving independently from various myoviridae phage and flexible (F-type) tailocins derived from a phage in the siphoviridae family (Ghequire and De Mot 2015; Hockett et al. 2015; Stice et al. 2023). Although a variety of bacteria besides pseudomonads have been shown to produce tailocins, much of our current knowledge concerning tailocin killing and resistance comes from studies investigating R-type tailocins (also known as R-type pyocins) of *P. aeruginosa* (Scholl and Martin 2008; Williams et al. 2008; Köhler et al. 2010; Scholl 2017; Patz et al. 2019; Ge et al. 2020). R-type pyocins have been shown to efficiently kill sensitive cells through direct lysis by mechanical disruption of target cell membranes, with binding of tailocins determined largely by receptor binding proteins (Rbps) and their associated chaperones (Williams et al. 2008; Köhler et al. 2010; Salazar et al. 2019). These 2 loci together appear to determine the targeting spectrum for all types of pseudomonad R-type tailocins examined, with the targeting spectrum for *P. syringae* R-type syringacins shown to rapidly evolve through localized recombination of regions containing the Rbps and chaperone (Baltrus et al. 2019).

The outer membranes of gram-negative bacteria are decorated with a variety of proteins and sugars, with one of the most well-known structures being the lipopolysaccharide (LPS) layer (Bertani and Ruiz 2018). The LPS is composed of covalently bound chains of various sugar moieties, the compositions of which vary within and between species, which are anchored into the outer membrane (King, Kocíncová, et al. 2009). In gram-negative bacteria such as *P. aeruginosa*, biosynthesis of these sugar moieties occurs in the cytoplasm of the cell independently for *O*-antigen and core LPS structures, with polymers initially built to differing lengths and compositions on the cytoplasmic face of the inner membrane. Sugar polymers for the core or *O*-antigen chains are then independently moved from the cytoplasmic face of the inner membrane onto the periplasmic face through multiple transport pathways, such as flippase enzymes or ATP-Synthase Binding Cassette (ABC) transporters, after which all components of LPS chains are connected. Lastly, the Lpt system positions LPS sugars in the outer membrane (Lam et al. 2011; Bertani and Ruiz 2018). Independent modifications and decorations can be added to the growing chains at multiple steps of the process by dedicated enzymes. Since the LPS is a major component determining interactions of bacteria with the immune systems of hosts, is often used for attachment of phage, and is likely involved in a variety of other processes important for survival across bacterial populations and communities, composition of its LPS is thought to be under strong diversifying selection and can be notoriously variable even between closely related strains (Lam et al. 2011). Earlier studies on the R-pyocins of *P. aeruginosa* implicated the LPS as the binding sites for tailocins and specifically by the Rbps and pointed toward either rhamnose or glucose as the interacting sugar moieties (Köhler et al. 2010). *P. aeruginosa* strains are able to produce multiple types of *O*-antigen that are potentially linked to the LPS core: one type of chain is referred to as the common polysaccharide antigen (CPA) or A band and is composed of repetitive chains of D-rhamnose linked together, while the other chain can be composed of more diverse sugars and is referred to as the *O*-specific-antigen (OSA) or B band (Pier 2007; King, Kocíncová, et al. 2009). Both the CPA and OSA are held in position in the outer membrane through their attachment to a conserved core chain of sugars. A simplified diagram showing biosynthesis and positioning of the CPA and OSA in Pseudomonas strains is provided as Supplementary Fig. 1.

Early studies pointed towards the outer core sugars of *P. aeruginosa* as binding sites for R-type pyocins, with the presence, composition, and length of these different *O*-antigen bands implicated as playing a role in shielding or enabling attachment of tailocins to LPS-associated sugars (Köhler et al. 2010; Heiman et al. 2022). Furthermore, RB-TnSeq across a handful of *P. fluorescens* strains implicated both the core and OSA/B band of the LPS as interaction sites for these molecules, with disruption of genes from all of these regions providing resistance against tailocin-based killing (Carim et al. 2021). Conversely, multiple papers have implicated the CPA or A band of the LPS as a main site of interactions of tailocins from *P. syringae*, as genetic data suggest that *P. syringae* strains may lack the OSA/B band and since deletions or modifications of genes within the CPA production pathway differentially affect tailocin targeting to otherwise sensitive strains (Hockett et al. 2017; Jayaraman et al. 2020). Indeed, in natural populations of *P. viridiflava* (classified within the *P. syringae* species complex and sharing vertically inherited tailocin structural loci with *P. syringae*), LPS conformation is correlated with tailocin sensitivity (Backman et al. 2024). This difference in which *O*-antigen band tailocins target is perhaps not completely surprising, as tailocins of *P. syringae* are derived from a different progenitor phage than the R-type pyocins of *P. aeruginosa* and many of the R-type tailocins of *P. fluorescens* (Hockett et al. 2015).

We have previously published a screen of tailocin killing and sensitivity across phylogenetically divergent *P. syringae* strains, where we were able to delineate tailocins into qualitatively different killing classes and strains into qualitatively different sensitivity classes based on their interactions. Specifically, we showed that killing class 1 tailocins can target strains in sensitivity class B, and conversely, that killing class 2 tailocins target strains in sensitivity class A. Here, we use this data to perform a genome-wide association study to map the genetic basis of R-type syringacin sensitivity across a variety of closely related *P. syringae* strains and identify genomic regions associated with differing sensitivity to these tailocins. We extrapolate from these results to map and predict tailocin sensitivity across a diverse range of isolates from this species, including numerous strains where LPS has been previously characterized, and uncover genomic trends that appear to enable prediction of tailocin sensitivity for these strains.

## Methods

### Genome sequences used for genome-association studies and tailocin prediction studies

All genomes referenced in this manuscript are listed in Table 1, which also lists accessions for these sequences at GenBank and abbreviations used for each strain in the manuscript. We additionally report draft and complete genomes for a variety of strains that have had their LPS conformations previously characterized across different reports (Zdorovenko and Zdorovenko 2010), as well as complete genome sequences for 2 closely related strains (CC1417 and CC1524) containing plasmids that contain numerous genes predicted to affect LPS biosynthesis. Statistics for genome sequencing and assembly for LPS-characterized strains, as well as CC1417 and CC1524, are described in Supplementary File 1 at https://doi.org/10.6084/m9.figshare.22688020. Pipelines and methods used for genome assemblies can also be found in Supplementary File 1 (Li 2016; Tatusova et al. 2016; Wick et al. 2017; Kolmogorov et al. 2019).

**Table 1.** Strains used in this study.

Strain	Abbreviation	Genome accession	Tailocin sensitivity group
*P. syringae* pv. *glycinea* A29-2	*Pgy* R4	ADWY00000000.1	A
*P. syringae* pv. *phaseolicola* 1644	*Pph* 1644	AGAS00000000.1	A
*P. syringae* pv. phaseolicola 1448a	*Pph* 1448a	GCA_000012205.1	A
*P. syringae* pv. *aesculi* 0893_23	*Pae*	AEAD00000000.1	A
*P. savastanoi* NCPPB 3335	*Psv*	GCA_000164015.3	B
*P. syringae* pv. *mori* MAFF301020	*Pmo*	AEAG00000000.1	B
*P. syringae* pv. *lachrymans* MAFF370057	*Pla* YM7902	GCA_030252675.1	B
*P. syringae* TLP2	TLP2	GCA_018516745.1	B
*P. syringae* CC1458	CC1458	AVEN00000000.2	A
*P. syringae* CC440	CC440	GCA_000452605.3	A
*P. syringae* pv. *japonica* MAFF301072	*Pja*	AEAH00000000.1	A
*P. syringae* pv. *atrofaciens* DSM50255	*Paf*	AWUI00000000.1	A
*P. syringae* CC457	CC457	AVEB00000000.2	B
*P. syringae* pv. *aptata* DSM50252	*Ptt*	AEAN00000000.1	A
*P. syringae* CC1543	CC1543	AVEJ00000000.2	A
*P. syringae* pv. *pisi* 1704B	*Ppi*	AEAI00000000.1	A
*P. syringae* pv. *syringae* B48	B48	GCA_030035225.1	A
*P. syringae* CC94	CC94	AVEA00000000.2	B
*P. syringae* USA011	USA011	GCA_000452525.4	B
*P. syringae* pv. *syringae* B301D	B301D	GCA_000988485.1	A
*P. syringae* UB303	UB303	GCA_000452565.3	B
*P. syringae* pv. *syringae* B728a	*Psy* B728a	GCA_000012245.1	A
*P. syringae* CC1629	CC1629	AVEE00000000.2	B
*P. syringae* CC1513	CC1513	AVEL00000000.2	B
*P. syringae* pv. *oryzae* 1_6	*Por* 1_6	GCA_000156995.2	B
*P. syringae* pv. *tomato* DC3000	*Pto* DC3000	AE016853.1	B
*P. syringae* pv. *lachrymans* MAFF302278	*Pla* 106	AEAM00000000.1	B
*P. syringae* pv. *tomato* T1	*Pto* T1	ABSM00000000.1	B
*P. syringae* CC1630	CC1630	AVED00000000.2	B
*P. syringae* CC1544	CC1544	AVEI00000000.2	B
*P. syringae* USA007	USA007	AVDY00000000.2	A
*P. syringae* CC1416	CC1416	AVEP00000000.2	B
*P. syringae* pv. *morsprunorum* MAFF302280	*Pmp*	AEAE00000000.1	A
*P. syringae* pv. *actinidiae* ICMP 18884	*Pan* ICMP 18884	GCA_000648735.3	A
*P. syringae* CC1559	CC1559	AVEG00000000.2	A
*P. syringae* CC1466	CC1466	AVEM00000000.2	B
*P. syringae* CC1557	CC1557	GCA_000452705.3	B
*P. cannabina* pv. *maculicola* ES4326	*Pma* ES4326	GCA_000145845.2	B
*P. asturiensis* CC1417	CC1417	GCA_000452825.3	A
*P. asturiensis* CC1524	CC1524	GCA_000452745.3	A
*P. syringae* pv. *helianthii* LMG 5067	*Phel* LMG 5096	GCA_022557235.1	Unscreened
*P. syringae* pv. *tagetis* IMCP 4091	*Ptg* ICMP 4091	GCA_022557255.1	Unscreened
*P. syringae* pv. *tomato* 2170	*Pto* 2170	JAPTOE000000000.1	Unscreened
*P. syringae* Cit7	Cit7	GCA_000145825.2	Unscreened
*P. syringae* pv *oryzae* 36_1	*Por* 36_1	GCA_001283935.1	Unscreened
*P. syringae* pv. garcae NCPPB 588	*Pga* NCPPB 588	JBGMSQ000000000.1	Unscreened
*P. syringae* pv. garcae NCPPB 2708	*Pga* NCPPB 2708	JBGMSU000000000.1	Unscreened
*P. syringae* pv. garcae NCPPB 1399	*Pga* NCPPB 1399	JBGMSS000000000.1	Unscreened
*P. syringae* pv. *ribicola* NCPPB 1010	*Pri* NCPPB 1010	JBGMSR000000000.1	Unscreened
*P. syringae* pv*. delphinii* NCPPB 1879	*Pdl* NCPPB 1879	JBGMST000000000.1	Unscreened
*P. syringae* pv*. lachrymans* NCPPB 1096	*Pla NCPPB* 1096	JBGMSV000000000.1	Unscreened
*P. syringae* pv*. tabaci NCPPB* 79	*Pta NCPPB* 79	JBGMSW000000000.1	Unscreened
*P. syringae* pv. tagetis ICMP 6370	*Ptg* ICMP 6370	JBGMSZ000000000.1	Unscreened
*P. syringae* pv. morsprunorum CFBP 1650	*Pmp* CFBP 1650	JBGMSY000000000.1	Unscreened
*P. syringae* pv. helianthi CFBP 1732	*Phel* CFBP 1732	JBGMSX000000000.1	Unscreened

### Genome-wide association with tailocin sensitivity

We downloaded .fasta sequences for each of the genomes in Table 1 that had been previously screened for tailocin sensitivity in Baltrus et al. (2019) and used Prokka to reannotate each in a consistent manner (Seemann 2014). We then carried out a clustering analysis of the .gff files from these genomes using Roary and with default parameters (Page et al. 2015). Last, we used Scoary to associate tailocin sensitivity with clusters of orthologous genes found by Roary (Brynildsrud et al. 2016). Tailocin sensitivity classes for each gene as used in the Scoary analysis are listed in Table 1, and we note that sensitivity classes for strains CC1630, *Pto*T1, and *Pla*106 differ from what was published in Baltrus et al. (2019) due to labeling errors in the previous manuscript. All input and output files for Prokka, Roary, and Scoary analyses can be found as Supplementary files at https://doi.org/10.6084/m9.figshare.22688020.

### Phylogenetic analysis

For each of the reported phylogenies, we obtained either nucleotide (*ychF* and *dctP*) or protein (RfbD) sequences from each of the genomes listed in Table 1 and aligned these using Clustal Omega with default parameters (Sievers and Higgins 2018). We used Modeltest to find the best evolutionary model describing this alignment (Darriba et al. 2020), which was either TIM2 + I + G4 (*ychF* and *dctP*) or JTT + G4 (RfbD). We then inferred phylogenetic relationships from these alignments using RaxML-ng (Kozlov et al. 2019). We used the following command to infer phylogenies and bootstrap support for *ychF*, *dctP*, and RfbD:

raxml-ng -msa (ALIGNMENT.FASTA) --prefix (PREFIX) --bs-metric fbp,tbe
with model specified as either --model TIM2 + I + G4 or --model JTT + G4

All input and output files for phylogenetic analyses can be found as Supplementary files at https://doi.org/10.6084/m9.figshare.22688020.

### Soft-agar overlay of previously unscreened strains for tailocin sensitivity

Soft-agar overlays were carried out as described previously (Hockett and Baltrus 2017). Briefly, overnight cultures of strains CC440, USA011, USA007, and CC1416 were diluted back 1:100 in King's B media (KB) and grown for 4 h at 27 °C while shaking at 220 rpm. At this point, mitomycin C was added to these cultures to a final concentration of 0.75 µg/mL and the cultures were incubated under the same conditions overnight. The next day, tailocins from these strains were purified from the supernatant by filtration through 0.22 µm syringe filters.

For overlays, strains of interest were grown overnight in KB media at 27 °C while shaking at 220 rpm. The following morning, cultures were diluted 1:100 in fresh KB media and incubated for an additional 4 h under the same environmental conditions. At this point, 100 to 300 µL of bacterial cultures (enough bacteria to yield a confluent layer in the soft agar in 1 d) was mixed with 3 mL of 0.4% molten agar and poured onto the top of a KB agar plate. Top agar was allowed to solidify for ∼10 min, at which point a 10 µL sample of each tailocin was added. Zones of clearing for each strain were observed and photographed either the next day or 2 d later, depending on the growth of the target strain. Overlay assays were performed at least 3 independent times, and representative results are reported. Unedited pictures of the overlay results shown in figures can be found in Supplementary files at https://doi.org/10.6084/m9.figshare.22688020.

## Results

### Genomic association for tailocin sensitivity across *P. syringae*

There have been numerous instances where closely related strains of *P. syringae* qualitatively differ in the phenotype of tailocin sensitivity (Baltrus et al. 2019). We therefore hypothesized that we could use our previously reported strain classifications for tailocin sensitivity, coupled with genomic analyses, to highlight strong and independent signals pointing to the identity of genes and pathways contributing to differential tailocin sensitivity. The results of an analysis using Roary and Scoary to correlate genomic changes with tailocin sensitivity are shown in Table 2, and we find that 17 different loci are significantly (*P* < 0.05) associated with tailocin sensitivity by these analyses and after Benjamini–Hochberg correction for multiple tests. A subset of 11 of these remain significantly associated with tailocin sensitivity after Bonferroni correction for multiple tests (*P* < 0.05). Differences in statistical significance due to decisions about the control of type 1 error, whether to control the probability of at least 1 error (the Bonferroni correction) or to control the proportion of type 1 errors (the false discovery rate) are expected (Benjamini and Hochberg 1995). In this case, the same genomic locations (between *dctP* and *ychF*) are identified with both approaches. We include both sets of statistical results to provide a complete picture of the Scoary analysis but emphasize that, regardless of the test used, all loci identified as significant in one or both analyses occur in the same genomic contexts across strains. Looking at the larger set more specifically, there are 9 loci whose presence is strongly correlated with tailocin sensitivity group A (as per Baltrus et al. 2019) and 8 whose presence is strongly correlated with tailocin sensitivity group B. Each of the 17 loci associated with each sensitivity group are co-located in syntenic genomic locations across strains, a location which corresponds to gene clusters implicated in production of the CPA chain of the LPS as per Jayaraman et al. (2020).

**Table 2.** Results from genome-wide association analysis.

Gene	Annotation in *Psy*B728a	Annotation in USA011	Annotation	# Class A present in (of 17)	# Class B present in (of 20)	Bonferroni corrected *P*-value	Benjamini–Hochberg corrected *P*-value
*rmd*	Psyr_0915	N/A	GDP-6-deoxy-D-talose 4-dehydrogenase	13	0	0.027422528	0.002492957
*gmd*	Psyr_0916	N/A	GDP-mannose 4,6-dehydratase	16	0	5.42E−05	1.35E−05
*wzm*	Psyr_0917	N/A	Sugar transport protein	15	0	0.000596142	8.52E−05
*rfbB*	Psyr_0926	N/A	dTDP-glucose 4,6-dehydratase	11	0	0.594154778	0.034950281
Glycosyltransferase	Psyr_0930	N/A	Putative glycosyltransferase	12	0	0.137112641	0.00856954
*prsD*	Psyr_0933	N/A	Type I secretion system ATP-binding protein	13	0	0.027422528	0.002492957
*prsE*	Psyr_0934	N/A	Type I secretion system membrane fusion protein	13	0	0.027422528	0.002492957
TolC-like	Psyr_0935	N/A	Outer membrane protein	14	0	0.004570421	0.000571303
*algA*	Psyr_0937	N/A	Alginate biosynthesis protein AlgA	16	0	5.42E−05	1.35E−05
*dapH*	N/A	N028_RS04635	2,3,4,5-tetrahydropyridine-2,6-dicarboxylate N-acetyltransferase	0	15	0.128170512	0.008544701
Glycosyltransferase	N/A	N028_RS04640	Putative glycosyltransferase	0	15	0.128170512	0.008544701
*wecE*	N/A	N028_RS04645	dTDP-3-amino-3,6-dideoxy-alpha-D-galactopyranose transaminase	0	15	0.128170512	0.008544701
ATPase	N/A	N028_RS04660	Type I secretion system ATPase	0	18	0.000487752	8.13E−05
Permease	N/A	N028_RS04665	ABC transporter permease	0	18	0.000487752	8.13E−05
*rfbC*	N/A	N028_RS04670	dTDP-4-dehydrorhamnose 3,5-epimerase	0	15	0.128170512	0.008544701
*rfbA*	N/A	N028_RS04680	Glucose-1-phosphate thymidylyltransferase	0	19	4.65E−05	1.35E−05
*rfbB*	N/A	N028_RS04690	dTDP-glucose 4,6-dehydratase	0	20	2.58E−06	2.58E−06

**Fig. 1.**
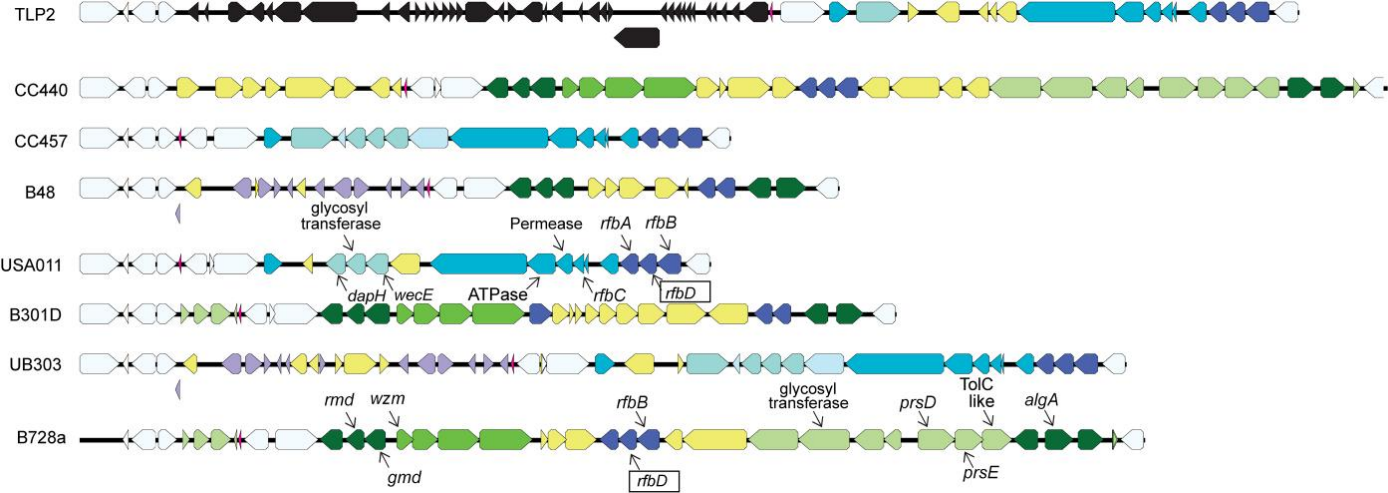
*O*-antigen/CPA locus of *P. syringae* phylogroup 2 strains. The genomic island found between *ildD-ychF* is shown for 8 phylogroup 2 strains. Strains are arranged vertically in phylogenetic order according to Baltrus et al. (2019) and were picked for the figure because they represent situations where closely related strains have switched tailocin sensitivity class phenotypes. Common loci not thought to be involved in LPS construction are shown in white, and loci that are part of a possible prophage in strain TLP2 are shown in black. Loci that are possibly involved in LPS construction but which are found across tailocin sensitivity class strains are colored purple, with stronger coloration indicating presence in a larger number of strains. Yellow loci are only found in single strains among the 8 shown. Loci that are possibly involved in LPS construction but which are found only within tailocin sensitivity class A and B strains are shown in green and blue, respectively, with stronger coloration indicating presence in a larger number of strains. Loci that were significantly associated with tailocin sensitivity class according to our analyses are labeled in strains *Psy*B728a (sensitivity class A) and USA011 (sensitivity class B), and we additionally label *rfbD* inside a box since we highlight its use as a predictor of tailocin sensitivity.

To demonstrate how this CPA locus could shift between closely related strains, we focused on investigating gene presence and synteny within the entirety of this region across a subset of phylogroup 2 strains (Fig. 1). This region is bracketed by loci conserved in every strain in phylogroup 2, with *ildD* coding for L-lactate dehydrogenase on one end and the gene for *ychF* coding for an ATPase on the other end. Genes found in this region in tailocin sensitivity group A include those involved in the production and modification of rhamnose moieties in the CPA (*gmd*, *rmd*, *wzm*, *algA*, and numerous glycosyl transferases and sugar modification enzymes). This region also encodes the transport extension and termination (TET) operon, an ABC transporter which moves CPA sugar chains from the cytoplasm to the periplasm, but as highlighted in Jayaraman et al. (2020) and elaborated on here, there is extensive sequence variation between these operons across sensitivity groups. Genes found in this region in tailocin sensitivity group B also include those involved in the production and modification of rhamnose moieties in the CPA (*rfbABC*, also known as *rmlABC*), as well as other glycosyl transferases and sugar modification enzymes and 2 potential secretion systems.

### *rfbD* as an indicator gene for tailocin sensitivity

**Fig. 2.**
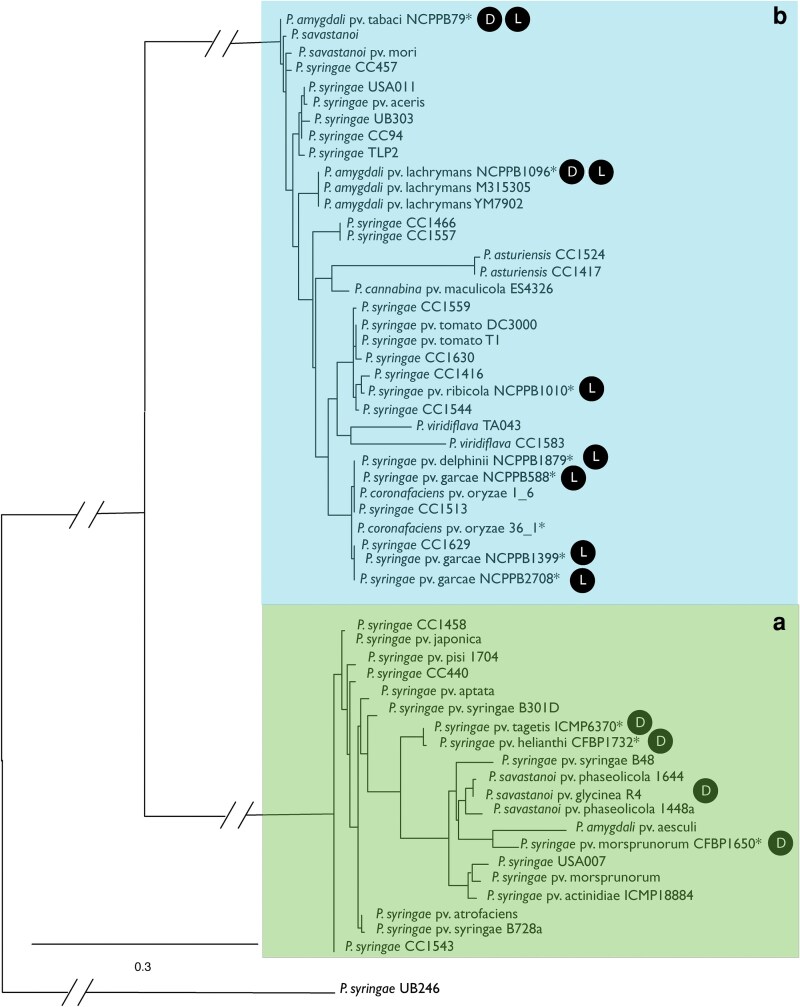
RfbD as a predictor of tailocin sensitivity classes in *P. syringae.* We inferred a maximum likelihood phylogeny from protein sequences of RfbD from across *P. syringae* strains tested for tailocin sensitivity in Baltrus et al. (2019) and from 16 additional strains which have had their genomes sequenced but which have not been previously screened. Strain names are shown matching abbreviations in Table 1. Clades containing strains previously typed as tailocin sensitivity classes A and B are colored green and blue, respectively. Alleles of RfbD from previously unscreened strains are designated in the phylogeny with *, and additionally, alleles from strains with previously characterized LPS conformations are noted with a black circle containing either “l” for L-rhamnose or “d” for D-rhamnose (Zdorovenko and Zdorovenko 2010). Phylogeny shown is the best tree collapsed, with bootstrap values shown for important nodes using values from the transfer bootstrap expectation. All input and results files for phylogenetic analysis can be found in the Supplementary data at https://doi.org/10.6084/m9.figshare.22688020.

Upon inspection of the CPA locus across tailocin sensitivity classes, we observed that *rfbD*, encoding dTDP-4-dehydrorhamnose reductase, appears to be the only gene conserved in the *ildD-ychF* region across all strains. However, unlike the TET operon throughout *P. syringae* identified in a previous report (Jayaraman et al. 2020), sequence similarity across alleles of *rfbD* is extensive enough to be clearly identified and analyzed across strains by sequence searches alone. We therefore pulled sequences for alleles of this locus from all strains from the main *P. syringae* phylogroups as described in Dillon et al. (2019) (phylogroups 1 to 6 and 10) and analyzed in our previous paper (Baltrus et al. 2019), as well as 5 strains for which tailocin sensitivity has yet to be screened and 10 strains for which LPS has been previously characterized but which also have yet to be screened for tailocin sensitivity (Zdorovenko and Zdorovenko 2010) and inferred a maximum likelihood phylogeny from these sequences. We rooted this phylogeny using the RfbD allele from strain UB246 because this phylogroup 13 strain is one of the most divergent within the *P. syringae* species complex and strains from this phylogroup are commonly used as outgroups (Baltrus et al. 2019; Dillon et al. 2019). As shown in Fig. 2, alleles of RfbD form 2 distinct clades, with clade membership showing a complete match to tailocin sensitivity in all previously screened strains. From this phylogeny, we predict that strains *Pto*2170, Cit7, *Por* 36_1, *Phel* LMG 5067, and *Ptg* ICMP 4091 would be characterized as sensitivity classes B, B, B, A, and A, respectively, based on *rfbD* sequences. Furthermore, all strains where the LPS has been previously characterized (Zdorovenko and Zdorovenko 2010), as dominated by D-rhamnose (*Pmp* CFBP 1650, *Ptg* ICMP 6370, and *Phel* CFBP 1732), are predicted to be tailocin sensitivity class A, while those dominated by L-rhamnose (*Pga* NCPPB 588, *Pga* NCPPB 1399, *Pga* NCPPB 2708, *Pri* NCPPB 1010, and *Pdl* NCPPB 1879) are predicted to be tailocin sensitivity class B. Lastly, 2 strains appear to maintain both L- and D-rhamnose in their LPS based on previous characterization (*Pta* NCPPB 79 and *Pla* NCPPB 1096), but their *rfbD* alleles clearly cluster with tailocin sensitivity class B. Despite this clear correlation, we do note that *rfbD* did not appear as a significant locus in our genome-wide association studies with tailocin sensitivity. We speculate that this lack of a result can be explained by the way in which Roary searches for clear presence–absence polymorphisms across clusters of orthologous genes instead of querying allelic variation within clusters. It is possible that divergent *rfbD* alleles were grouped as orthologs in these analyses by the software pipelines due to sequence similarity and thus were not highlighted for significant correlations with tailocin sensitivity across strains.

**Fig. 3.**
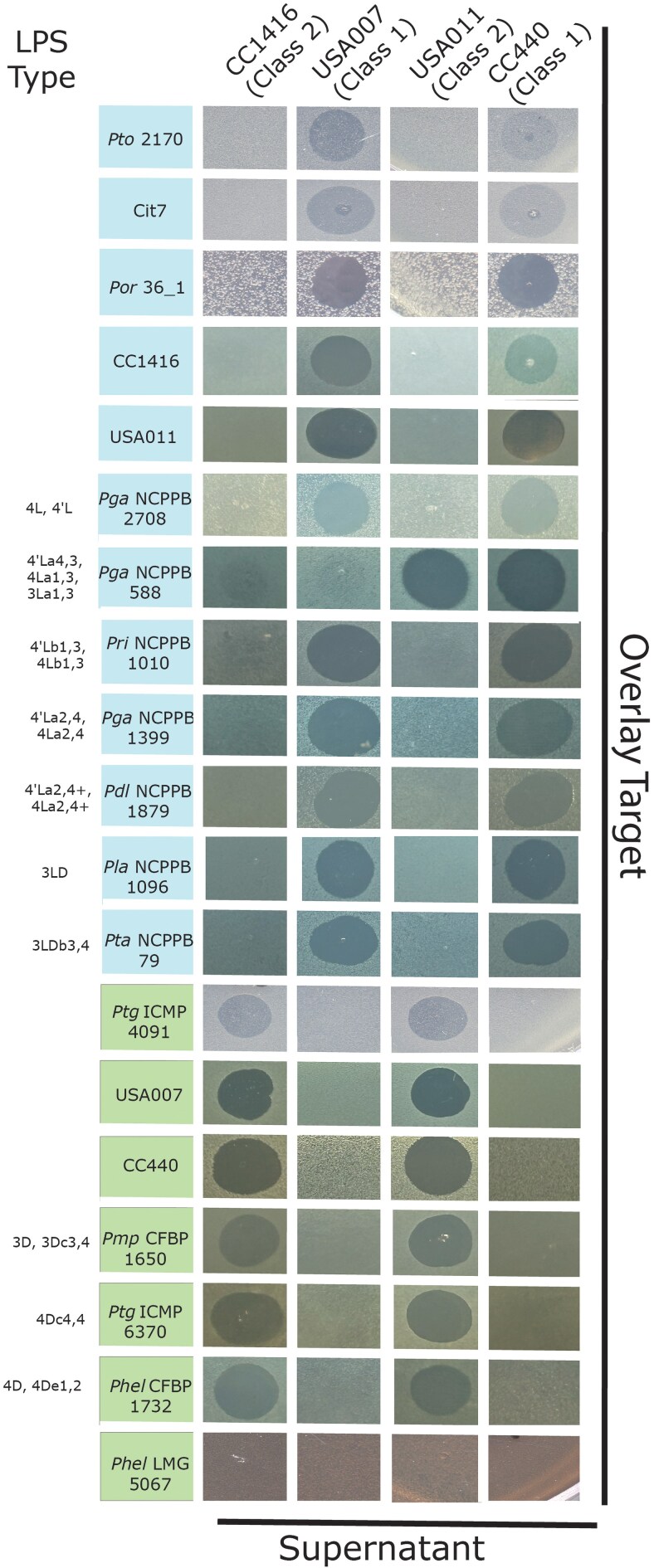
Tailocin prediction for unscreened strains largely matches predictions. We performed soft agar overlay assays where tailocins from 4 different strains and 2 different phylogenetic groups of *P. syringae* (USA007 and CC440, tailocin killing class 1, blue boxes; CC1416 and USA011, tailocin killing class 2, green boxes) were tested against 16 strains for which tailocin sensitivity has not been previously reported, as well as against themselves (strains listed in the *Y* axis, with strain names colored for predicted tailocin sensitivity class as per phylogeny in Fig. 2a). Strain names are abbreviated as per Table 1. When strains being tested for sensitivity have had their LPS characterized, we list these characterizations to the left of their names with nomenclature shown according to Zdorovenko and Zdorovenko (2010). For these characterizations, “l” indicates L-rhamnose moieties while “d” indicates D-rhamnose moieties, with numbers and denoting linkage of these sugars.

In our previous paper (Baltrus et al. 2019), we showed that tailocin-producing strains of *P. syringae* (and therefore presumably tailocins if each strain produces 1 tailocin) could be split into at least 2 distinct killing classes based on reactions of target strains. Furthermore, we showed that *P. syringae* strains could be grouped into at least 2 different sensitivity classes based on susceptibility to killing by tailocins. Thus, we established a framework whereby killing class 1 tailocins target sensitivity class B strains and killing class 2 tailocins target sensitivity class A strains. Here, we performed overlay assays to characterize tailocin killing across 16 strains mentioned above, which were not included in our previous tailocin sensitivity screen (Fig. 3). Instead of an all by all screen as we previously performed, we used a set of 4 strains that include phylogenetically independent representatives from each tailocin killing class: CC440 (phylogroup 2, tailocin killing class 1), USA011 (phylogroup 2, tailocin killing class 2), USA007 (phylogroup 3, tailocin killing class 1), and CC1416 (phylogroup 3, tailocin killing class 2). Results from these overlays largely follow predictions from *rfbD* phylogenies as *Ptg*ICMP4091, *Pmp*CFBP1650, *Ptg*ICMP6370, and *Phel*CFBP1732 are classified as sensitivity group A (light green box). Likewise, Cit7, *Por* 36_1, and *Pto*2170, *Pga*NCPPB2708, *Pri*NCPPB1010, *Pga*NCPPB1399, *Pdl*NCPPB18979, *Pla*NCPPB1096, and *Pta*NCPPB79 are classified as sensitivity group B (light blue box). We saw no visible tailocin activity against PhelLMG5067 using these 4 indicator tailocin strains, nor from screening this strain with tailocins from a larger collection of bacterial isolates (Supplementary Fig. 2). In sum, when tailocin activity is witnessed, all strains where LPS has been previously shown to be dominated by D-rhamnose are sensitivity class A, while those dominated by L-rhamnose are sensitivity class B, with 1 exception (*Pga*NCPPB 588).

To better characterize the evolutionary history of this LPS biosynthesis cluster throughout *P. syringae* in a broader context than phylogroup 2 strains, we searched for syntenic loci surrounding this region throughout the genomes of all strains listed in Table 2. Although this region is bordered by *ilvD* and *ychF* in phylogroup 2 strains, we found that the position of *ilvD* is not conserved throughout all phylogroups of the species and thus focused on *dctP* as an alternative locus bordering this region throughout the species (although we note that *dctP* is not found in phylogroup 9 strains). Phylogenies inferred using nucleotide sequences of *dctP* and *ychF* for all strains are consistent with vertical inheritance of these loci as strains largely form consistent clades for both loci that match phylogroup structures as well as phylogenies inferred from whole genomes and multi-locus sequence typing studies (Supplementary Fig. 3). These clades are denoted by coloration of each phylogroup overlayed onto the phylogenies as per our previous publications, and we use this coloration to highlight that strains for each phylogroup form consistent groupings. Within this figure, we also recolor the protein phylogeny for RfbD as per phylogroup structure, which demonstrates a relatively high amount of intermixing of strains from different phylogroups within independent clades of RfbD and a lack of vertical inheritance of this locus (Supplementary Fig. 3).

### Numerous predicted LPS biosynthesis and modification genes found on a plasmid in phylogroup 9 strains

**Fig. 4.**
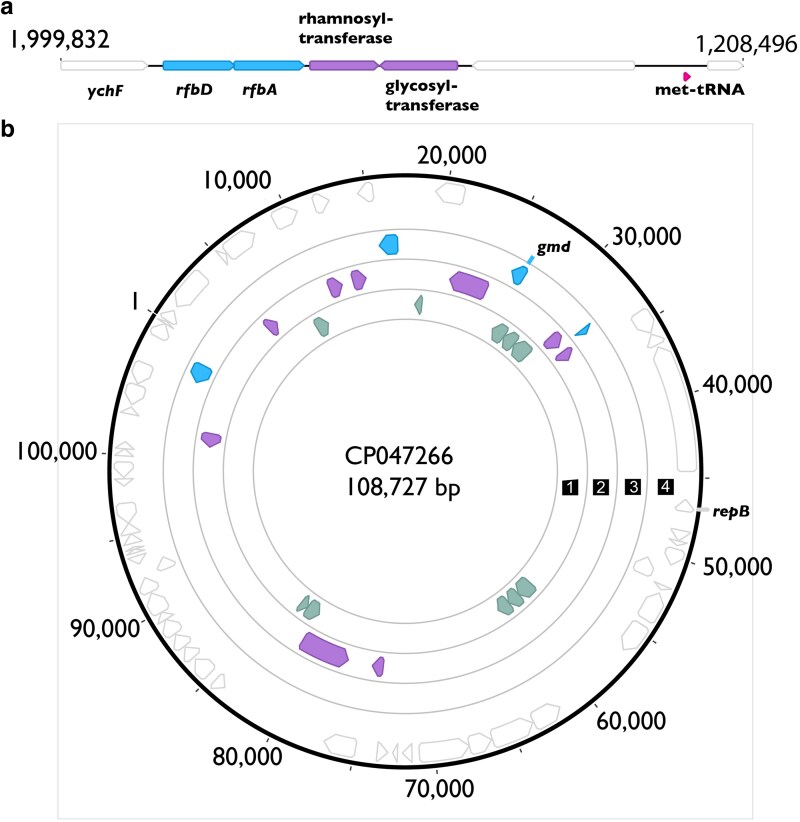
Potential for plasmid-encoded LPS biosynthesis pathways in phylogroup 9 strains. a) A stylized diagram showing the reduced CPA locus for *P. syringae* phylogroup 9 strains CC1417 and CC1524. This locus is bordered by *ychF* on one side (as with all other genomes). Since there was no clear orthologue of either *dctP* or *ilvD* in the vicinity of this locus for these genomes, we denote a border which brackets this locus, along with *ychF*, by showing a tRNA-Met locus which is found in proximity to the CPA locus across *P. syringae* genomes. Loci of interest are listed, with genomic positions in the CC1417 genome listed at the top of the diagram. b) A diagram of the potential LPS-modifying plasmid of *P. syringae* CC1417. GenBank accession for this plasmid is listed in the middle, along with the total size. Genes found on this plasmid are shown in 4 concentric rings, with rings representing the following annotations: (i) Potential type I secretion system genes, (ii) glycosyl and other sugar transferases, (iii) other LPS-associated biosynthesis, and (iv) genes with no clear LPS association. We further highlight the position of *gmd* (which plays a key role in the biosynthesis of D-rhamnose for LPS incorporation), as well as *repB* (a plasmid replication initiation gene).

Upon further inspection of genomes, we found that the main LPS biosynthesis region found in the chromosome of both phylogroup 9 strains, CC1417 and CC1524, is relatively reduced compared to all other strains reported within this manuscript. Indeed, in both strains this region contains only *rfbAD* and a small number of rhamnosyl/glycosyl transferases (Fig. 4a). We further found that both complete genome sequences for these strains also contain a circular plasmid containing numerous LPS modification genes, including the only predicted copy of *gmd* within these genomes, multiple components for type I secretion systems and ABC transporters, and 8 different glycosyltransferases (Fig. 4b).

## Discussion

Composition of the LPS layer of the outer membrane has been shown to impact multiple phenotypes crucial for the survival of bacteria that are widely distributed throughout the environment (Bertani and Ruiz 2018). Likewise, since the LPS often forms the interface between bacteria and hosts, changes in the LPS can have widespread effects during infection of plants by phytopathogenic bacteria (Newman et al. 2000; Meyer et al. 2001). Our findings support previously published results implicating the LPS, and specifically a structure analogous to the CPA chain of *P. aerguinosa* formed by rhamnose polymers, as a primary target of *P. syringae* tailocins. Previous results suggested that changes in LPS composition across strains of pathovar *actinidae* were due to localized recombination of allelic variation of the TET operon (Jayaraman et al. 2020), which is part of this CPA locus. While our results echo these previous reports, the patterns reported here skew more toward the presence/absence of genes and broad structural differences in the arrangement of the operons and a much larger region of recombination involved (∼20 kb) rather than redistribution of allelic variation in a subset of these genes (Supplementary Fig. 3). We further evaluate each of these operons in the context of LPS production below.

### *gmd, rmd*, and *algA*

Alleles of *gmd* and *rmd* are differentially present within the CPA locus of strains classified as sensitivity class A (represented by strain *P. syringae* pv. *syringae* B728a, *Psy*B728a, in Fig. 1) and form orthologous clusters according to the Roary pipeline, which are significantly associated with tailocin sensitivity according to Scoary. As shown in Supplementary Fig. 1, together with *wbpZ*, these genes are predicted to form an operon involved in the biosynthesis and linkage of D-rhamnose in the *O*-antigen of the LPS (Jayaraman et al. 2020).

D-rhamnose in the LPS is formed through the production of guanosine diphosphate (GDP)-D-rhamnose from GDP-D-mannose. *gmd* is predicted to encode GDP-mannose 4,6-dehydratase, an enzyme that carries out the first step in this pathway and converts GDP-D-mannose to GDP-4-dehydro-6-deoxy-D-mannose (aka GDP-6-deoxy-d-lyxo-hexos-4-ulose) (King et al. 2009). *rmd* is predicted to encode GDP-6-deoxy-d-lyxo-hexos-4-ulose-4-reductase, which produces GDP-D-rhamnose from the substrate produced by Gmd (King et al. 2009). *wbpZ* is a glycosyltransferase that can attach D-rhamnose to the growing LPS chain from GDP-D-rhamnose (Wang et al. 2015). Alleles of AlgA are encoded by genes within the CPA locus in strains from *P. syringae* tailocin sensitivity class A, and *algA* is predicted to encode a bifunctional protein with phosphomannose isomerase and GDP-mannose pyrophosphorylase activities, which catalyze the conversion of fructose-6-phosphate to mannose-6-phosphate and mannose-1-phosphate to GDP-mannose (Rocchetta, Pacan, et al. 1998). It is likely that this enzyme leads to the production of the substrate (GDP-mannose) that is acted upon by Gmd, encoded in the CPA locus, significantly associated with tailocin sensitivity in class A strains. Notably, in contrast to strains from all other phylogroups, we report here that *gmd* appears to be plasmid encoded in both phylogroup 9 strains (CC1417 and CC1524). Disruption of this operon in the sensitivity class A strain *P. syringae* pv. *actinidiae* ICMP 18884, and specifically disruption of *gmd* and *wbpZ*, has been shown to provide resistance to tailocin killing (Hockett et al. 2017). It has also been shown that a point mutation in *rmd* can provide tailocin resistance in the sensitivity class A strain *P. syringae* pv. *phaseolicola Pph* 1448a (Kandel et al. 2020).

### *rfbA*, *rfbB*, and *wecE*

Alleles of *rfbA*, *rfbB,* and *wecE* are differentially present within the CPA locus of strains classified as sensitivity class B (represented by strain *P. syringae* USA011, in Fig. 1).

In contrast to the D-rhamnose biosynthesis pathway described above (and where D-rhamnose is linked to a GDP intermediate form prior to incorporation in the LPS), biosynthesis of L-rhamnose occurs through a distinct pathway and is ultimately linked to a deoxythymidine diphosphate (dTDP) intermediate prior to LPS incorporation. RfbA (also known as RmlA) is a glucose-1-phosphate thymidylyltransferase, which catalyzes the formation of dTDP-glucose from dTTP and glucose 1-phosphate (Rocchetta, Burrows, et al. 1998). RfbB is a dTDP-glucose 4,6-dehydratase, which catalyzes the dehydration of dTDP-D-glucose to form dTDP-6-deoxy-D-xylo-4-hexulose (Rocchetta et al. 1999). WecE is a dTDP-4-amino-4,6-dideoxygalactose transaminase (Hwang et al. 2004). It should be noted that there are 2 distinct enzymes (RfbA and RffH) identified in *Escherchia coli* that can carry out this same reaction, and that these enzymes function in different pathways and are present in different operons in *E. coli* (Marolda and Valvano 1995; Sivaraman et al. 2002). Since dTDP-L-rhamnose is the donor molecule for L-rhamnose incorporated into the LPS, and each of these enzymes is predicted to be involved stepwise production of dTDP ultimately linked to L-rhamnose, it is likely that these enzymes play a key role in creation of L-rhamnose that can subsequently be incorporated into the *O*-antigen of tailocin sensitivity class B strains.

Lastly, an independent orthologous cluster of *rfbB* alleles is also significantly associated with tailocin sensitivity class A strains. While this set of genes likely encodes an enzyme with similar functionality to those that are found in sensitivity class A strains, there is significant divergence between alleles of *rfbB* from each sensitivity class.

### ABC transporters and related loci (*wzm*, ATPase, *prsD*, *prsE*, TolC-like, *rfbC*, and permease)

For class A genomes, the presence of an apparent orthologue of *wzm* is significantly correlated with tailocin sensitivity. In *P. aeruginosa,* rhamnose precursors of the CPA are transported from the cytoplasm of the cell by an ABC transporter (Wzt/Wzm), with energy provided by an ATPase, and with a fourth glycosyltransferase gene present in the operon to attach these sugars to the growing chain (Jayaraman et al. 2020; Warring et al. 2022). This operon in *P. syringae* is the same as highlighted by Jayaraman et al. (2020) for its patterns of recombination, and it is likely that this operon in tailocin sensitivity class A strains is carrying out the same activities as the *wzt/wzm* ABC transporter in *P. aeruginosa*. Also worth noting is that there is a fourth gene encoding a glycosyltransferase (likely *wbdD*), which appears to be part of this operon in sensitivity class A genomes. However, significant divergence for this gene within sensitivity classes due to recombination likely leads to a lack of overall significant associations for this enzyme with class A strains, as pointed out by Jayaraman et al. Disruption of this operon, and specifically within *wzm, wzt,* and *wbdD* has been shown to provide resistance to tailocin killing of the sensitivity class A strain *P. syringae* pv. *actinidiae* ICMP 18884 (Hockett et al. 2017; Jayaraman et al. 2020).

Class B genomes potentially encode multiple transporters that each contain subsets of genes significantly associated with tailocin sensitivity. One of these transporter operons appears to encode 4 genes that likely encode an ABC transporter used by Jayaraman in phylogenetic analyses as a predictor of tailocin sensitivity (Jayaraman et al. 2020) (and which is analogous to the potential *wzt/wzm* ABC transporter system mentioned above but divergent both in sequence and in annotation). Three of these genes in this operon are significantly associated with tailocin sensitivity for class B: a permease, *rfbC* (dTDP-4-dehydrorhamnose 3,5-epimerase), and an ATPase. As with the *wzt/wzm* operon mentioned above, there is often a fourth glycosyltransferase gene downstream of these 3, but which is divergent enough across strains so that significant associations with tailocin sensitivity are likely obscured.

One additional ABC transporter is significantly associated with tailocin sensitivity in many class A genomes, and contains genes similar to *prsD*, *prsE*, as well as a locus potentially encoding a TolC-like protein. Unlike the 2 transporter operons mentioned above, there does not appear to be a glycosyltransferase as part of this operon across strains. PrsD and PrsE are involved in exporting acidic polysaccharides in *Rhizobium* as part of biofilm formation, with PrsD acting as an ATP-binding protein and PrsE as a membrane fusion protein (Russo et al. 2006), but their functions within these *P. syringae* strains are unknown. TolC is a membrane channel protein for type I secretion systems (Vakharia et al. 2001).

### Glycosyltransferases

Sugar chains of the LPS are built through the action of glycosyltransferases, which take sugars linked to a variety of substrates and transfer them to the growing LPS chains (Cote and Taylor 2017; Whitfield et al. 2020). One glycosyltransferase, potentially found in an operon with *wecE* mentioned above, is significantly correlated with sensitivity group B strains. One glycosyltransferase is significantly associated with sensitivity class A strains and appears to be present as part of a larger operon across these strains; however, it is the only gene from this operon that shows a significant association. Aside context clues, such as other enzymes encoded in operons with these loci, it is difficult to pinpoint the functions of these glycosyltransferases without further experiments. We also note that there appear to be multiple glycosyltransferases on plasmids of phylogroup 9 strains CC1417 and CC1524.

### dapH

*dapH* putatively encodes 2,3,4,5-tetrahydropyridine-2,6-dicarboxylate *N*-acetyltransferase, which catalyzes the transfer of an acetyl group from acetyl-CoA to tetrahydrodipicolinate (Beaman et al. 1998) and which can be involved in the production of bacterial structures like peptidoglycan. There are multiple copies of similar enzymes throughout the *P. syringae* genome, but this specific copy is correlated with sensitivity class B genomes.

Previous manuscripts demonstrated that allelic variation within the TET operon could be effectively used for the prediction of tailocin sensitivity (Jayaraman et al. 2020) across some strains; however, the TET operon shares little sequence similarity across strains within different tailocin sensitivity classes. Thus, there may not be an easy way to design probes to identify sensitivity classes without whole genomes from the TET operon alone. Our analyses further identify *rfbD* as a potential additional candidate gene for extrapolating and predicting tailocin sensitivity classes from genome sequence alone for most *P. syringae* strains. Upon examination of the CPA locus of numerous *P. syringae* genomes, and unlike many of the genes significantly associated with tailocin sensitivity listed in Table 2, we observed that alleles of *rfbD* were always present within this island, even though these alleles did not rise to the level of significance in the genome-wide association study. Phylogenetic inference of amino acid sequences for RfbD clearly shows that alleles associated with each sensitivity group cluster with each other, and to the exclusion of sequences from the alternative sensitivity group, with strong support. Furthermore, this placement enabled relatively high precision in assigning previously unscreened strains to sensitivity classes (Figs. 2 and 3). Although RfbD appears critical for the creation of L-rhamnose (Lerouge and Vanderleyden 2002; Nian et al. 2007), a main component of the CPA chain of the LPS, it is unclear why there are 2 different phylogenetic clades for these genes that map cleanly to *P. syringae* tailocin sensitivity classes and whether both phylogenetic clades of RfbD have similar functionalities for the cell.

LPS composition is extremely variable among bacterial isolates, as past studies have focused on using LPS characterization (in part) for classification of different lineages of *P. syringae* (Zdorovenko and Zdorovenko 2010), but relatively few studies have evaluated the genomic basis of LPS diversity across *P. syringae* (sensu lato). Moreover, previous results have suggested that pseudomonads are somewhat unique in the bacterial world in that they have been shown to synthesize both L- and D-rhamnose to decorate structures on their outer membrane (Mishra and Balaji 2019). Our results here, coupled with previous characterization of LPS structures across a subset of these strains (Zdorovenko and Zdorovenko 2010), suggest that presence/absence diversity driven by relatively large-scale (∼20 kb) recombination events can dramatically and repeatedly alter LPS composition between closely related isolates and that such changes likely drive differences in sensitivity to different classes of tailocins. While the genetic basis of LPS configuration in *P. syringae* differs from canonical pathways established in *E. coli*, *P. aeruginosa*, and other systems, particularly in that the *O*-antigen appears to be dominated by enantiomers of rhamnose rather than a mix of variable sugar chains in addition to rhamnose, it remains unknown how such variation impacts recognition of these strains by the plant immune system. It also remains unknown what correlated phenotypic changes, in the context of behaviors like biofilm formation or in responses to LPS-interacting molecules like cationic antimicrobials (Huszczynski et al. 2020), might occur across *P. syringae* strains with fundamentally different LPS conformations. It is also highly likely that phage attachment and infection also greatly contribute to, and may indeed itself drive, modification of the LPS under natural conditions (Meaden et al. 2015; Warring et al. 2022). Our results highlight the potential for tailocins, or other LPS-targeting bacteriocins (McCaughey et al. 2014), to be used as tools for uncovering and exploring genetic diversity in pathways and genes that underlie LPS production in these and other strains.

Our results further demonstrate that, while the regions surrounding LPS biosynthesis loci in *P. syringae* are vertically inherited, the LPS biosynthesis locus itself appears to undergo repeated recombination events in which at least 2 different conformations are completely swapped (Figs. 1 and 2 and Supplementary Fig. 3). These events are reminiscent of localized recombination events for tail fiber genes within the tailocin biosynthesis cluster, and indeed, a recent publication builds on these results to demonstrate strong correlations between tail fiber structures and alleles of *rfbD* (and therefore LPS locus conformations) (Fautt et al. 2025). We also acknowledge that genetic variants of *rfbD* and the TET operon are often highly correlated due to these large-scale recombination events and that both loci therefore likely serve as effective predictors of tailocin sensitivity across a majority of *P. syringae* genomes. Toward this point, it is highly possible that investigation of the TET operon offers additional granularity for prediction of tailocin sensitivity if this locus is subject to additional recombination events above and beyond the relatively large events that swap *rfbD* alleles, as demonstrated previously (Jayaraman et al. 2020). In the context of these results, it may be surprising to some that tailocin tail fiber genes do not appear as significantly associated with sensitivity groups in our genome-wide association results reported earlier. One potential explanation for this apparent discrepancy is that, while almost all *P. syringae* strains we have analyzed are sensitive to at least one killing class of tailocins, a comparatively high percentage of strains do not appear to produce active tailocins due to potentially inactivating mutations (Hockett et al. 2015; Baltrus et al. 2019). Therefore, alleles for tail fibers from strains with “broken” tailocin loci might not cleanly match their sensitivity background because there is likely no selection on LPS structures to avoid self-killing. Furthermore, our previous research suggests higher levels of genotypic and phenotypic diversity in tailocin tail fibers than in tailocin sensitivity (e.g. there are at least 3 and likely more than 3 distinct tailocin killing classes), and this higher level of diversity could potentially obscure associations between tail fiber genes and sensitivity class (Baltrus et al. 2019).

There are 2 strains, *Phel*LMG5067 and *Pga*NCCBP588, for which predictions from *rfbD* alleles do not cleanly match with observed tailocin sensitivity. In the case of *Phel*LMG5067, phylogenetic relationships predict that this strain would be sensitivity class A, while we observed that it was resistant to all 4 tailocins (Fig. 3) and a larger collection of tailocins (Supplementary Fig. 2). This result is rare but not unprecedented and was seen with *P. syringae* pv. *aceris* in our previous manuscript (Baltrus et al. 2019). One potential explanation for such results is that both of these strains possess additional LPS-modifying enzymes that alter the composition of either the main or side chains of LPS in a manner that blocks tailocin binding (Köhler et al. 2010; Bertani and Ruiz 2018). *Pga*NCCBP588 is predicted to be in sensitivity group B, but is sensitive to supernatants produced by USA011 and CC440 in the results reported here. In our hands, and inconsistently across multiple assays, we have witnessed variability for this strain in tailocin sensitivity (data not shown), with various combinations of killing classes and strains showing activity. In a similar vein, there are 2 strains for which LPS characterization has shown to maintain LPS with both L- and D-rhamnose (*Pta*NCCBP79 and *Pla*NCCBP1096). In the assays presented, we show that both strains are clearly categorized as sensitivity group B despite having both rhamnose moieties within the LPS. While this was not demonstrated in the 3 assays underlying data in Fig. 3, we note that we occasionally (but not consistently) see clear killing activity from both tailocin classes against these and related strains from pathovars tabaci and lachrymans (e.g. in a small minority of assays, both CC1416 and USA011 tailocins display activity against strains from these 2 pathovars specifically, but not against other sensitivity class B strains outside the assays reported in this manuscript, data not shown). Thus, we speculate that there exists a subset of strains which maintain the genetic potential to encode LPS chains that sensitize them to killing by a variety of tailocin types, but that LPS conformation within these strains displays plasticity affected by additional undefined environmental or growth conditions that lead to variability in results. We acknowledge that while this level of variability in results for these strains is quite interesting from a biological perspective, and certainly worthy of follow-up experiments, these results are outliers since a far majority of strains tested do not demonstrate this level of variability in our hands.

Herein, we also report that at least 2 closely related phylogroup 9 strains (CC1417 and CC1524) maintain a unique configuration of LPS biosynthesis and modification genes compared with all other *P. syringae* genomes. All other strains referenced within this manuscript have at least a single dominant locus for LPS biosynthesis bordered by *ychF*, these 2 phylogroup 9 strains also maintain a ∼100 kb secondary replicon containing numerous genes whose annotations suggest potential to impact LPS modification, as well as a predicted plasmid replication gene but no conjugation machinery. Plasmid-encoded genes within these strains include *gmd* (coding for a critical enzyme for synthesis of D-rhamnose) as well as numerous glycosyltransferases and potential ABC transporters (Fig. 4). Although predicted LPS modification genes can often be found in mobile elements such as plasmid and prophage, this phylogroup 9 replicon is notable both for the proportion of predicted LPS biosynthesis genes compared with other mobile elements within *P. syringae,* as well as because, to our knowledge, *gmd* is found on the chromosome in all other strains. It is currently unclear how encoding of potential LPS-modifying genes by plasmids shifts evolutionary and ecological dynamics of target strains in the context of LPS-interacting tailocins, phages, and other antimicrobials. However, even though the phylogroup 9 plasmids do not appear to contain genes for conjugation, one prediction is that the presence of such genes on mobile elements allows rapid alteration of “resistance” mechanisms to these particles through dissemination of LPS-altering genes if mobilized by other means. It is also notable, although potentially just a coincidence, that the phylogroup 9 strains are also the only *P. syringae* strains which appear to completely lack the genetic capacity to produce tailocins (Baltrus et al. 2019).

In sum, our results shed light on the potential genetic basis of fundamental shifts in LPS conformation in the *P. syringae*, which appear to be strongly correlated with shifts in sensitivity to phage-derived bacteriocins. They suggest that differences in binding of 2 previously identified tailocin killing types could in part be due to differential binding of rhamnose enantiomers that form the basis of the *O*-antigen of the LPS of this phytopathogen, with tailocins in class 1 targeting L-rhamnose while those in class 2 target D-rhamnose. Toward this, we also highlight that a recent publication built upon these results to demonstrate that structural variation in tail fibers of *P. syringae* tailocins correlates strongly with tailocin sensitivity and with *rfbD* alleles across strains, showing that “long” tail fibers are associated with class 1, while “short” tail fibers of class 2 tailocins (Fautt et al. 2025). Lastly, our study points toward additional genomic loci that can be used to predict LPS and tailocin phenotypes from genomic sequences, providing a clear proof of concept for combining large-scale phenotypic screens of tailocin sensitivity with genome-wide association studies to identify the basis of bacteriocin sensitivity across strains.

## Supplementary Material

jkaf203_Supplementary_Data

## Data Availability

Strains and plasmids are available upon request. The authors affirm that all data necessary for confirming the conclusions of the article are present within the article, figures, and tables. The datasets and uncropped images generated during and/or analyzed during the current study are available in FigShare at https://doi.org/10.6084/m9.figshare.22688020. All genomic assemblies and raw reads for assemblies are available at GenBank at accession numbers specified either in Table 1 or in Supplementary Table 1 (found at FigShare at https://doi.org/10.6084/m9.figshare.22688020). Supplemental material available at *G3* online.
