# Supplementary material for: Genomic correlates of tailocin sensitivity in Pseudomonas syringae
Source: G3 (Bethesda). 2025 Aug 29;15(11):jkaf203. doi: 10.1093/g3journal/jkaf203 (PMC12608077; doi:10.1093/g3journal/jkaf203)
Supplement: jkaf203_Supplementary_Data [file jkaf203_supplementary_data.zip › Supplementary_Figure_Legends_G3-2025-406018.docx]

**Supplementary Figure 1. A Simplified Overview of LPS Biosynthesis and Transport in Pseudomonads.** Biosynthesis of D-rhamnose and L-rhamnose takes place in the cytoplasm, where the end products prior to LPS incorporation into the CPA are each rhamnose molecule linked to a nucleotide diphosphate intermediate (dTDP for L-rhamnose and GDP for D-rhamnose). Relevant enzymes as mentioned in the manuscript are named for each pathway. LPS sugar chains are independently built for both the core and O-antigen on the cytoplasmic face of the inner membrane, can be transported to the periplasm through ABC transporters (often but not always), and then transported to the outer membrane. We note that *P. syringae* appears to only produce a CPA O-antigen, but other Pseudomonads also produce a much more variable chain called the OSA.

**Supplementary Figure 2. Lack of Tailocin Killing Against Strain PhelLMG5067 Using a Broader Collection of Tailocins.** We carried out independent overlay assays against strains USA011, CC440, and PhelLMG5067 using a broader collection of tailocins than in the primary manuscript. Tailocins and overlay strains were treated and prepared in the same way as described in manuscripts with 10 μL samples of tailocin applied to confluent top agar containing potential target strains. Overlay strains are labelled left to right as follows: USA011, CC440, PhelLMG5067. Tailocins used are as follows: 1) TLP2 2) UB303 3) CC440 4) CC457 5) Pja 6) Pmo 7) CC1543 8) CC1630 9) USA01110) CC1416 11) Pla106 12) CC1544 13) Ptt 14) 15) PsyB728a 16) PsyB728a tailocin- mutant in which positive regulation of the tailocin locus has been disrupted (referred to as ΔR_reg_ in Hockett et al. 2015 (Hockett et al. 2015)).

**Supplementary Figure 3. The CPA Locus (and RfbD) Undergoes Extensive Recombination Across Strains*.* A)** A schematic showing general genomic context for regions of interest in *P. syringae* chromosomes. B) Phylogenies for *dctP, ychF,* and RfbD. ßWe inferred maximum likelihood phylogenies for nucleotide sequences from two loci which bracket the CPA locus across strains of interest in this manuscript and also include a recolored phylogeny RfbD across *P. syringae* strains from Figure 2. Strain names are shown matching abbreviations in Table 1. We have placed alleles of each strain in a colored box representing associations with various *P. syringae* phylogroups as defined by both MLST and whole genome studies and following our coloration scheme from previous manuscripts for these phylogroups. With the color scheme, presence of consistent color shading across strains within a phylogeny (as seen for both *dctP* and *ychF*) indicates consistent grouping based on MLST and whole genome phylogenies and thus vertical inheritance of the loci of interest. Repeated intercalation of colored boxes in multiple phylogenetic clades (RfbD) indicates horizontal gene transfer of these alleles within and across different phylogroups. Phylogenies shown are the best tree collapsed. All files for phylogenetic analysis can be found in the supplemental data at <https://doi.org/10.6084/m9.figshare.22688020.v4>
