# Supplementary figures and images for: Genomic correlates of tailocin sensitivity in Pseudomonas syringae
Source: G3 (Bethesda). 2025 Aug 29;15(11):jkaf203. doi: 10.1093/g3journal/jkaf203 (PMC12608077; doi:10.1093/g3journal/jkaf203)

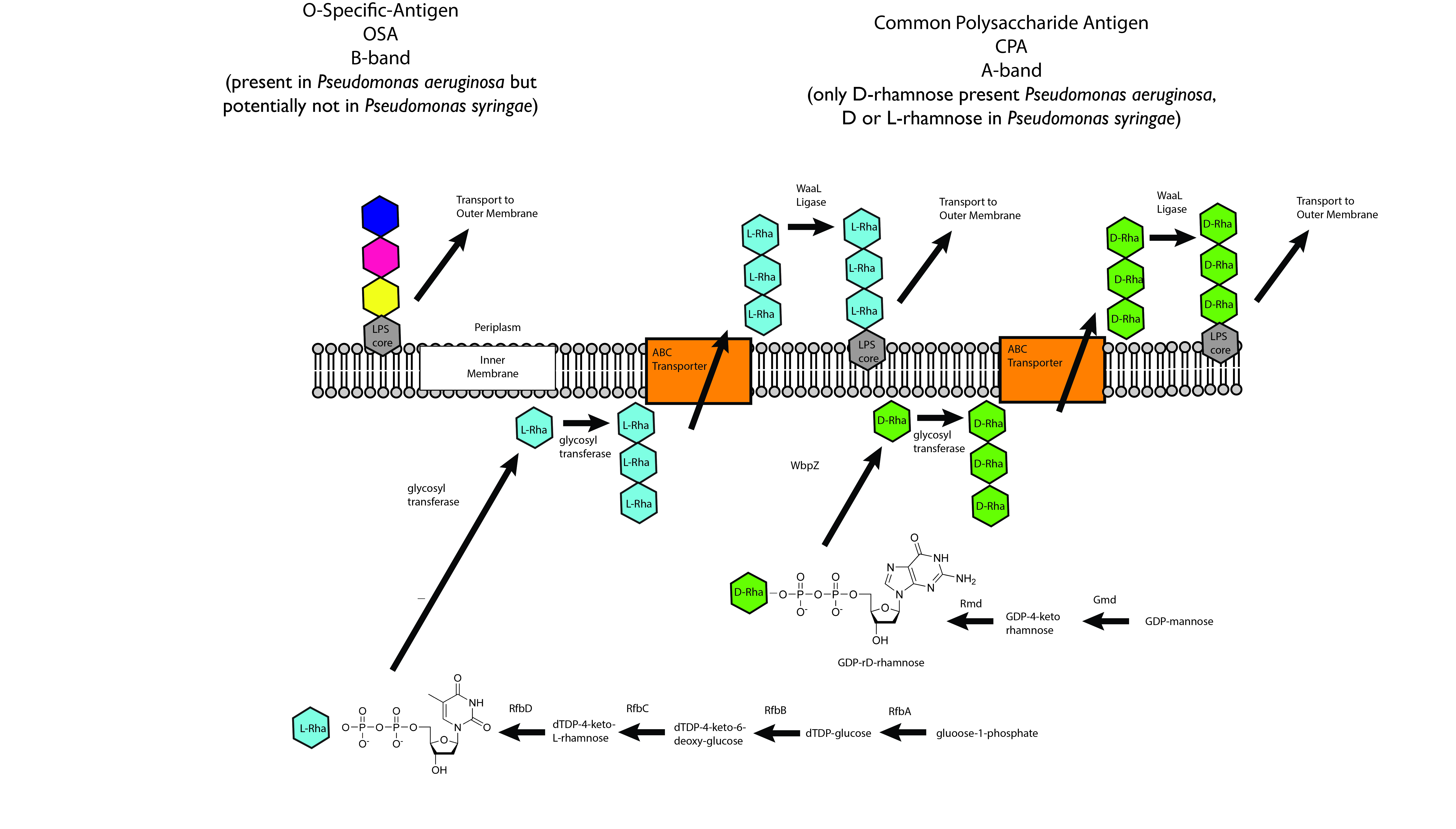

Supplement: jkaf203_Supplementary_Data [file jkaf203_supplementary_data.zip › Supplementary_Figure_1_G3-2025-406018.jpg]

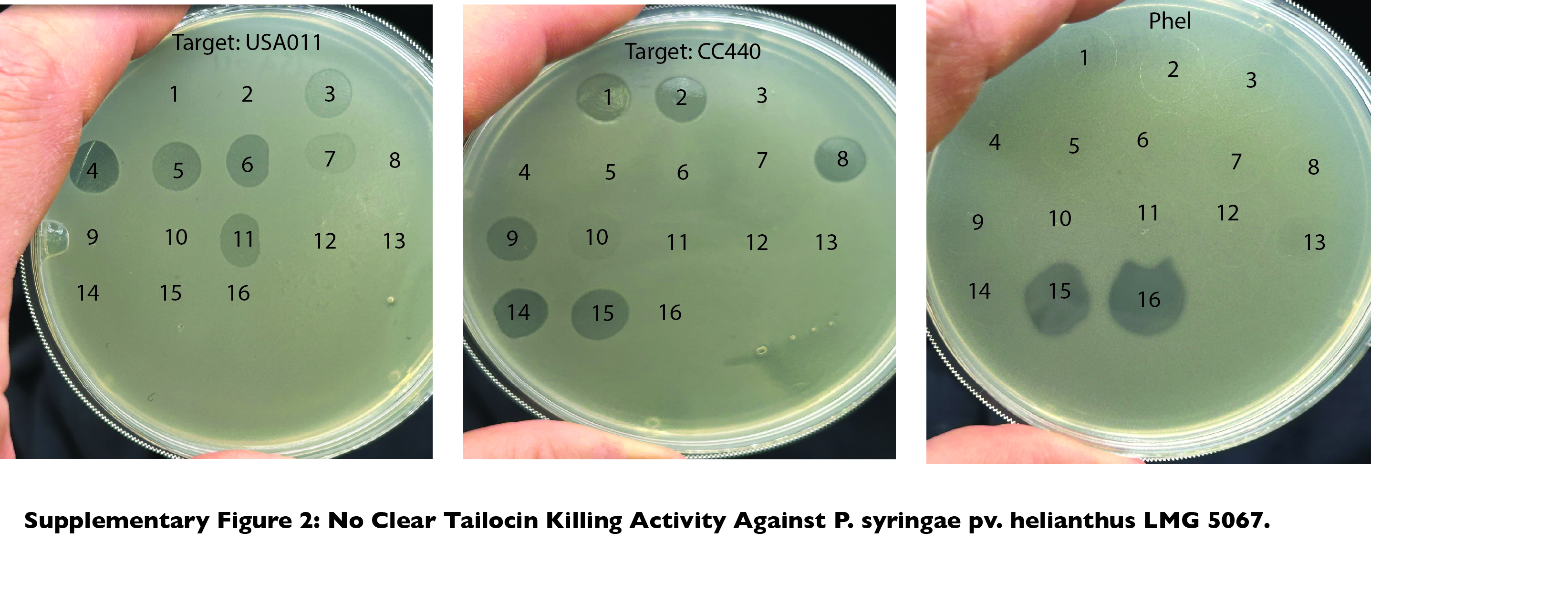

Supplement: jkaf203_Supplementary_Data [file jkaf203_supplementary_data.zip › Supplementary_Figure_2_G3-2025-406018.jpg]

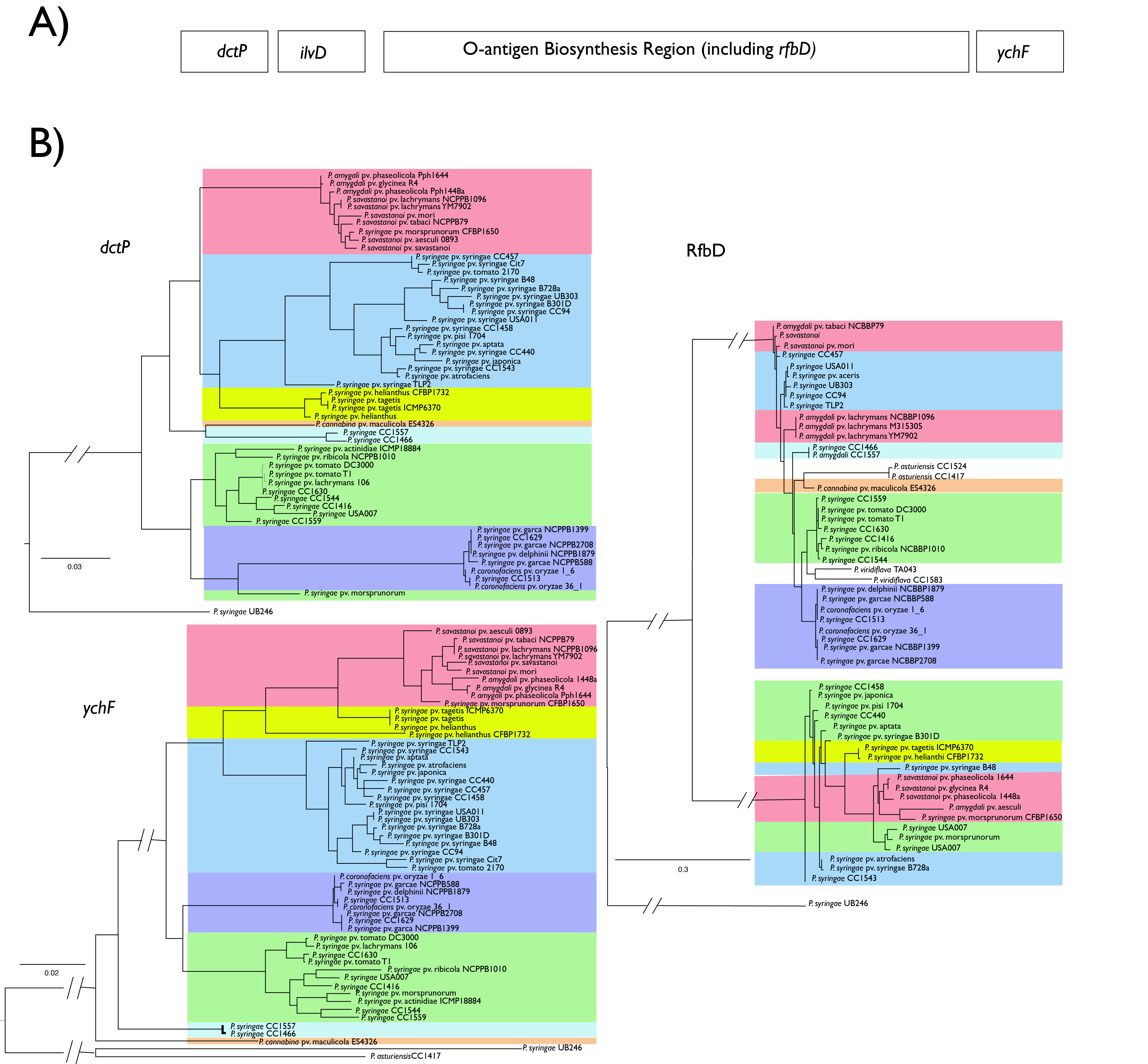

Supplement: jkaf203_Supplementary_Data [file jkaf203_supplementary_data.zip › Supplementary_Figure_3_G3-2025-406018.jpg]
